# Spatial Structure Facilitates Cooperation in a Social Dilemma: Empirical Evidence from a Bacterial Community

**DOI:** 10.1371/journal.pone.0077042

**Published:** 2013-10-22

**Authors:** Felix J. H. Hol, Peter Galajda, Krisztina Nagy, Rutger G. Woolthuis, Cees Dekker, Juan E. Keymer

**Affiliations:** 1 Department of Bionanoscience, Kavli Institute of Nanoscience, Delft University of Technology, Delft, The Netherlands; 2 Institute of Biophysics, Biological Research Centre of the Hungarian Academy of Sciences, Szeged, Hungary; 3 Instituto de Ecología y Biodiversidad, Santiago, Chile; Universidad Carlos III de Madrid, Spain

## Abstract

Cooperative organisms are ubiquitous in nature, despite their vulnerability to exploitation by cheaters. Although numerous theoretical studies suggest that spatial structure is critical for cooperation to persist, the spatial ecology of microbial cooperation remains largely unexplored experimentally. By tracking the community dynamics of cooperating (*rpoS* wild-type) and cheating (*rpoS* mutant) *Escherichia coli* in well-mixed flasks and microfabricated habitats, we demonstrate that spatial structure stabilizes coexistence between wild-type and mutant and thus facilitates cooperator maintenance. We develop a method to interpret our experimental results in the context of game theory, and show that the game wild-type and mutant bacteria play in an unstructured environment changes markedly over time, and eventually obeys a prisoner’s dilemma leading to cheater dominance. In contrast, when wild-type and mutant *E. coli* co-inhabit a spatially-structured habitat, cooperators and cheaters coexist at intermediate frequencies. Our findings show that even in microhabitats lacking patchiness or spatial heterogeneities in resource availability, surface growth allows cells to form multi-cellular aggregates, yielding a self-structured community in which cooperators persist.

## Introduction

Communities of cooperative organisms sharing limited resources or contributing to a common good are ubiquitous in nature, yet are vulnerable to disruption by free riders [1], [2]. The fact that cooperation is simultaneously ubiquitous and fragile, begs for an explanation regarding its emergence and maintenance. Microbes have been observed to cooperate in many different scenarios ranging from the sharing of public goods [3]–[7], to resource use [8], [9], the production of bacteriocins and virulence factors [10]–[13], and biofilm formation [14], [15]. Cooperative phenotypes can generally be maintained when cooperating individuals benefit more from cooperative acts than non-cooperators (cheaters), a situation referred to as assortment [16]. Spatial structure can be a critical determinant of the outcome of competitive interactions between cooperators and cheaters by facilitating assortment between cooperative individuals. Extrinsic ecological factors, e.g. spatial variations in resource availability, can give rise to a population structure that supports cooperator-cheater coexistence; while even in a spatially homogeneous environment, the intrinsic ecological dynamics of a community – such as birth, death, attachment, and dispersal – can result in a spatially structured population. Theoretical studies indicate that in a spatially structured yet otherwise homogeneous habitat, cooperators and cheaters may self-organize into a structured community that facilitates coexistence of the two types [17]–[20]. Despite the wealth of theoretical efforts, the spatial ecology of microbial cooperation remains largely unexplored experimentally. Here, we investigate the community dynamics of cooperator and cheater *Escherichia coli* in various habitats that differ in spatial structure and interpret our results in a game-theoretic framework.

*E. coli* bacteria collectively restrain growth to avoid population collapse when resources become scarce [21]–[23]. The collective decision to restrain growth *before* all resources are depleted is a form of altruistic cooperation, and one can think of the entry into stationary phase as a social contract: a collective rule of density-dependent growth regulation [9], [24]. The long-term benefits of going into stationary phase are evident: by limiting population growth, bacteria ensure that enough resources will be available to maintain a viable population for an extended period, and survive until conditions improve and growth can be resumed. In the short term however, ‘cheater’ cells that ignore the collective decision to restrain growth and that utilize the scarce resources to proliferate instead of investing in maintenance, may increase in numbers. As a result, cheaters jeopardize the survival of the collective – which in turn could face a tragedy of the commons [25].

In *E. coli*, this social contract is implemented through the switching of sigma factors. Sigma factors enable RNA polymerases to address distinct parts of the genome to adequately cope with changing environments [23], [26]. The *rpoS* gene encodes for the starvation/stationary-phase sigma-factor 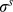, which outcompetes the main exponential-phase housekeeping 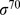 when resources run low, guiding the cell into stationary phase [27]. In late stationary-phase batch-cultures, mutants deficient in 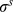 typically emerge after 8 to 10 days [9], [28], [29] and have been shown to take over wild-type (WT) *E. coli* populations [28]. These mutants exhibit a growth advantage in stationary phase (GASP) phenotype that, in case of the *rpoS819* mutant used in this study, is caused by an attenuated functioning of 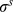 [29]. Attenuation of 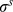 allows GASP mutants to cheat by ignoring social cues to inhibit growth, and instead continue a lavish lifestyle [9]. Thus, GASP mutants continue to proliferate, while *rpoS* WT bacteria invest energy in enhancing stress resistance and maintain a constant population size in which birth and death rates are balanced. By squandering resources and delaying stationary phase, GASP mutants defect on the social contract in which WT cooperate.

Game theory can be applied when different types employ different strategies in social interactions [30], [31] and has proven a useful framework to interpret the community and evolutionary dynamics of social microorganisms. The use of game theory has provided insight into the sugar metabolism of *Saccharomyces cerevisiae* [4]–[6], coinfection of viruses [32], [33], fruiting body development of *Myxococcus xanthus* [34], and stationary-phase entry and resource use of *E. coli* [9]. In the realm of game theory, cells ‘play’ a certain strategy resulting in a ‘payoff’ that translates into fitness. The two growth strategies encoded in the *rpoS* gene of *E. coli*, can be interpreted in a game-theoretic framework [9]: when cooperators (*rpoS* WT) meet cooperators, both inhibit growth and get rewarded (receiving payoff 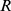) by allocating resources necessary for population maintenance; cooperators facing cheaters (*rpoS819*) get cheated upon as their savings get consumed prematurely, hence cooperators receive the sucker’s payoff 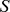; cheaters meeting cheaters get punished (payoff 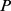) by growing to unsustainable numbers and jeopardizing long-term viability; while cheaters facing cooperators are tempted (payoff 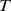) to exploit cooperators. By estimating these payoffs, predictions can be made about the long-term community composition and its stability.

Here, we study the community dynamics of *rpoS* WT cooperators and *rpoS819* GASP mutants in various habitats that vary in spatial structure. We first quantitatively describe the community dynamics in a simple well-mixed ecological scenario and develop a method to interpret the WT-GASP interaction in a game-theoretic framework. In order to explore the influence of spatial structure on cooperator-cheater competition we performed competition experiments in microfabricated habitats. Time-lapse fluorescence microscopy allows us to characterize WT-GASP community dynamics in habitats that are structured at the micrometer scale and address whether cooperators can persist in the presence of cheaters.

## Results and Discussion

### GASP Mutants Behave as Cheats

**Figure 1:**
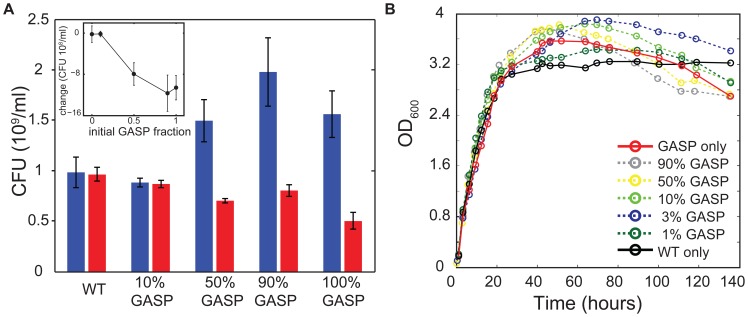
GASP mutants initially reach a high population density and subsequently decrease population viability. (**a**) Colony forming units (CFU) measured at day 1 (blue bars) and day 4 (red bars) of pure WT and GASP cultures and co-cultures with starting fractions of 90% WT and 10% GASP, 50% WT and 50% GASP, and 10% WT and 90% GASP. Error bars indicate the standard error of the mean of three replicate cultures. The inset shows that the change in the number of CFUs between day 4 and day 1 depends on the initial GASP fraction of a culture. (**b**) Growth curves, measured as optical density at 600 nm, of well-mixed batch cultures. A pure WT culture (black) sustains its population density for days, whereas a pure GASP culture (red) initially reaches a higher population density which later declines and drops below the level of the pure WT culture. WT-GASP co-cultures (dashed lines) show the frequency dependence of the overshooting and subsequent decline of population density.

We began by validating that *rpoS819* GASP mutants behave as cheaters and defect on the social contract of density-dependent regulation, by determining the amount of culturable cells in well-mixed cultures (LB medium, shaken flasks) starting from various WT/GASP ratios after one and four days of growth. Figure 1A shows that after one day of growth (blue bars), a pure GASP culture reaches a significantly higher density (measured by colony forming units) than a pure WT culture (two-sample t-test, 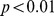); co-cultures consisting of equal starting fractions of WT and GASP or starting from a GASP majority (90%) also reach a significantly higher population density when compared to a pure WT culture (two-sample t-test, 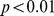). After four days of growth (red bars), the pure WT culture and 10% GASP co-culture both have sustained their population density (no change in viability counts between day 1 and 4; two-sample t-test, 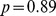 and 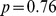, respectively). In contrast, cultures that reached a higher density at day one, suffered a higher mortality in the following days and show a significantly decreased viability at day four (two-sample t-test, 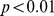). The inset in Figure 1A shows that the loss of viability between day one and four is higher for cultures that had a larger initial fraction of GASP cells. These results demonstrate that although defection of the social contract by the GASP mutant initially results in a larger population, it jeopardizes the survival of the collective in the long run – hence, GASP cells cheat [9].

Typical growth dynamics are represented in Fig. 1B, showing the population density of well-mixed cultures starting from various WT/GASP ratios measured by optical density. These curves illustrate that a pure WT culture reaches its maximum density earlier than a pure GASP culture that continues growth and reaches a higher maximum. In contrast to the pure WT culture that maintains a constant density for days, the density of the GASP culture starts to decline as soon as the maximum is reached. Cultures inoculated with a mix of WT and GASP *E. coli* (dashed lines Fig. 1B) indicate that the overshooting and subsequent decline of population density depends on the initial GASP fraction.

### Determining Game Dynamics from Experimental Data

Depending on the payoffs for cooperating and cheating, the dynamics of a cooperator-cheater community can be characterized as a certain game (e.g. a prisoner’s dilemma or a snow-drift game) and predictions about the community dynamics can be made [1]. Using the temporal changes in the relative abundance of the two types we can infer which game governs the community dynamics by estimating the fitness (relative growth rate) of a single cheater cell in a cooperator majority and vice versa. We base our approach on a two-type replicator equation [35] for the fraction of cheaters, 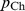, present in the population:
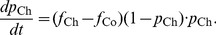
(1)


Note that the fraction of cooperators 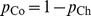. Linear approximations of the frequency-dependent fitnesses for cooperators, 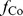, and cheaters, 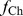, are
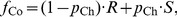
(2)

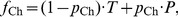
(3)respectively. Where the payoffs for cooperators (Co) and cheaters (Ch) are represented by:



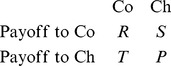
(4)The net fitness advantage of cheaters relative to cooperators, 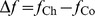, determines the change of the cheater fraction and in this case is given by:
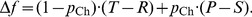
(5)


If we envision a lonely cheater in a majority of cooperators, i.e. 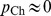, the second factor of the right-hand side of equation 5 vanishes, whereas by contrast the first factor cancels for a cheater majority (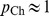). In these two limits we can thus express the payoff differences as the per capita growth rate of the cheater fraction by substituting Eq. 5 into Eq. 1 for the two different limits taken:
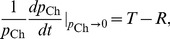
(6)

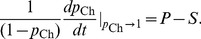
(7)


Because we are only interested in the limits of 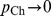 and 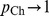, the assumption of linearity of Eqs. 2 and 3 can be relaxed without changing the results of our analysis.

This frequency-dependent fitness landscape 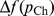 is parameterized by the payoff differences between temptation and reward 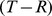 and between punishment and sucker payoff 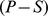. Interestingly, only the sign of the payoff differences needs to be obtained to identify which game is being played, while the actual values of the payoffs are less important. Once the payoff hierarchy is determined, the community dynamics can be classified into a game. For instance, when both 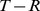 and 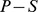 are positive the community adheres to a prisoner’s dilemma which in a well-mixed system results in cheater dominance, whereas cooperator-cheater coexistence would be possible in the snow-drift regime (where 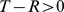 but 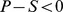).

### GASP Outcompete WT in an Unstructured Environment

**Figure 2:**
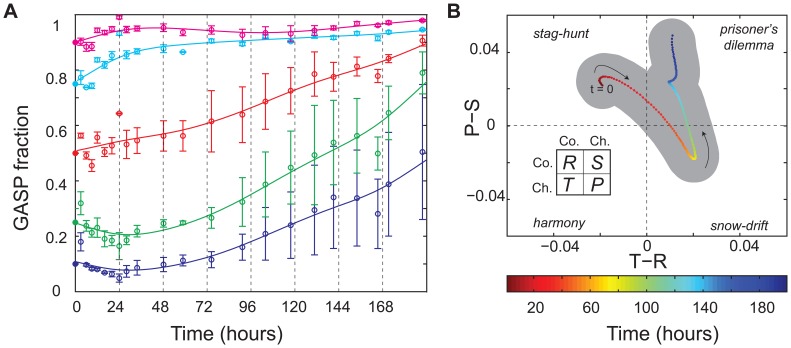
Long-term WT-GASP coexistence is not stable in well-mixed cultures. (**a**) Fraction of GASP cheaters (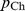) through time in well-mixed cultures measured by flow cytometry of GFP labelled WT and RFP labelled GASP cells. Circles mark means of three independent batch cultures (error bars indicate SEM) with initial GASP fractions of 0.9 (magenta), 0.75 (cyan), 0.5 (red), 0.25 (green) and 0.1 (blue); initial GASP fractions were obtained by mixing appropriate dilutions of WT and GASP mono-cultures (measured by optical density) and are represented with dots. Eventually, the GASP fraction increases in all cultures, regardless of initial GASP fraction. (**b**) Temporal game-dynamics of WT-GASP competition. The x-axis represents the payoff differences between Temptation and Reward, the y-axis represents the difference between the Punishment and Sucker's payoff; the four quadrants correspond to the four possible two-player games (inset: payoff matrix). Points are colored according to time and show that competition initially adheres to a stag-hunt game, briefly resembles a snow-drift game and finally settles as a prisoner's dilemma. The shaded area depicts the variation between experiments (standard deviation of replicates).

To gain insight into WT-GASP community dynamics in an unstructured environment, we used flow cytometry to monitor the relative abundance of neutrally labelled *rpoS* WT *E. coli* and *rpoS819* GASP mutants for 8 days in continuously shaken flasks. Figure 2A shows the time course of the GASP fraction (number of GASP cells/total number of cells) of independent competition experiments which each had five different starting fractions of GASP and WT cells. Experiments for each starting fraction were performed with three independent replica cultures. To obtain the payoff differences we analyze these data as described above, by estimating the fitness (relative growth rate) of a single GASP mutant cell in a WT majority and vice versa (see Materials and Methods). Figure 2B organizes the results from this analysis in 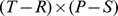 payoff space and shows that the game that WT and GASP bacteria play is not static, but changes over time.

During the first 24 hours of co-culture, the relative abundance of the cheater strain decreases for cultures starting from GASP fractions 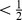, whereas the opposite is true for cultures starting from a cheater majority, as can be seen from Fig. 2A. This dependence on initial GASP fraction is the signature of a stag-hunt game [36]. In a stag-hunt game the initially dominant strategy takes over [37], suggesting that in the early phase of the experiment it benefits to be among individuals carrying the same genotype. It was previously shown that loss of *rpoS* function not only influences stationary-phase physiology, but also affects the expression of genes involved in several metabolic functions, chemotaxis, motility and several other functions during earlier phases of growth [38], [39]. It is possible that differences in exponential-phase physiology affect WT-GASP co-cultures in a frequency dependent manner, giving an advantage to the strain that is the majority. Such dynamics could lead to a stag-hunt game, however, a more extensive characterization of differences in WT and GASP physiology in early phases of growth would be necessary to shed light on the mechanism that makes WT and GASP initially interact according to a stag-hunt game.

During the first day of WT-GASP co-culture, the environment changes dramatically as resource levels decrease and metabolic products build up. Consequently, the GASP mutant starts to defect on the social contract: it delays entry into stationary phase and sustains growth at the expense of WT cells. The temptation to defect thus gradually increases and after 24 hours it becomes bigger than the reward for cooperation (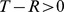), turning the community dynamics from a stag hunt into a prisoner’s dilemma (PD) game. In a PD game, the relative abundance of mutants increases in all cultures, irrespective of the GASP fraction. On day 3 and 4, the difference between the punishment and suckers payoff (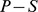) decreases and the community is at the boarder between a prisoner’s dilemma and a snow-drift game: GASP cells increase in fraction in cultures where they were the minority initially while their population share remains approximately constant in cultures where they were the vast majority initially. From day 5 on however, the punishment evidently becomes larger than the sucker’s payoff (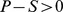) and the community dynamics are again governed by a PD: the GASP fraction increases in all cultures regardless of its relative abundance (see Fig. 2). This indicates that in the long run WT cooperators are expected to go extinct in an unstructured environment, and GASP mutants may reach fixation.

The fact that payoffs depend on the environment and therefore can change through time has been under appreciated in previous game-theory studies concerning microbes [5], [9], [32], [33], although it may have significant consequences for the outcome of cooperator-cheater competition. Our results show that a change as simple as a decrease in the abundance of resources over time, may not only influence the intensity of a competitive interaction (i.e. the magnitude of payoff differences) but can also change the underlying competitive hierarchy (i.e. the sign of payoff differences).

The WT-GASP community commences as a stag-hunt game, which opens up the possibility for cooperators to drive cheaters extinct if sufficiently abundant initially. Under the conditions investigated, however, cheaters were not sufficiently rare to be outcompeted by cooperators. If, in contrast to a batch culture, a habitat would have a steady in- and out-flow of nutrients and waste, conditions resembling earlier phases of the well-mixed batch culture could be maintained. In such a setting, community dynamics could stabilize as a stag-hunt game resulting in different competitive outcomes. This scenario might be applicable to natural ecosystems with a constant nutrient flux, and could be assessed experimentally by use of continuous-culture techniques.

### WT and GASP Coexist in Spatially Structured Microhabitats

Next, we turn to the influence of spatial structure on WT-GASP community dynamics. In order to test whether WT and GASP *E. coli* indeed can coexist (i.e. persist in culture together in the long-term) in a spatially structured but otherwise homogeneous environment, we performed competition experiments (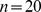) in microhabitats consisting of 85 connected habitat patches (see Fig. S3). Microhabitats are spatial ecosystems that mimic the fine-scale spatial structure of natural bacterial habitats, and allow bacteria to develop into a spatially structured metapopulation (a group of interacting subpopulations) [40], [41]. Because bacteria inhabiting a microhabitat can switch between planktonic (free swimming) and sessile (surface associated) lifestyles, a dynamically structured community in which cells aggregate in biofilms but also disperse from those, emerges in an initially homogeneous environment.

We used epi-fluorescence microscopy to track the community dynamics of WT and GASP cells at 15-minute intervals. The abundance of both strains was measured by converting raw fluorescence images into occupancy data which reports the area that a strain occupies (see materials and methods). Measuring occupancy rather than fluorescence intensity is less sensitive to differences in brightness of the two fluorescent proteins (GFPmut2 and mRFP) and the differential influence of environmental factors such as medium conditioning and population density on those proteins. To negate any remaining bias towards one of the reporters, half of the experiments were performed with GFP-labelled WT and RFP-labelled GASP strains, whereas the other half of the experiments were performed with a WT-GASP pair in which the fluorescent reporters were swapped – yielding equivalent results (see Fig. S4D and E for an analysis of these two datasets seperatly).

**Figure 3:**
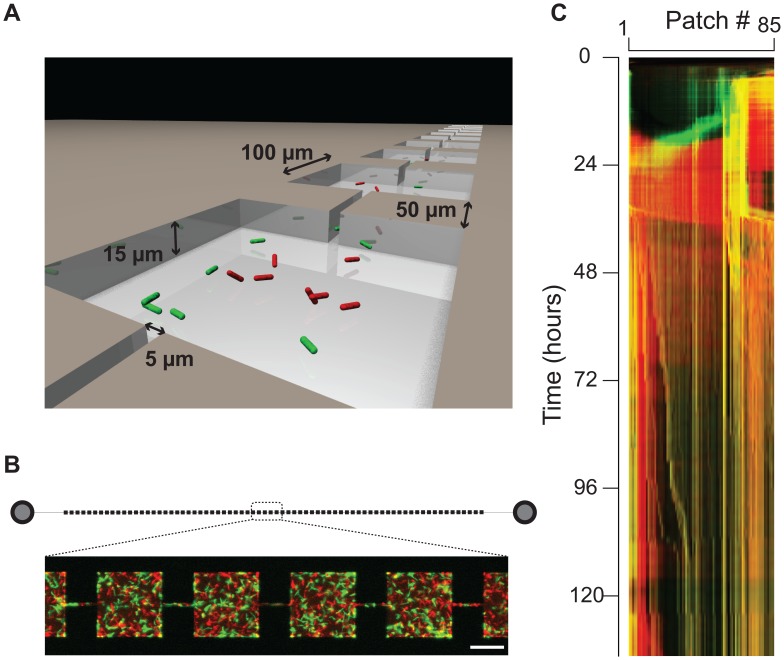
WT-GASP community in a microhabitat. (**a**) Schematic view of a microhabitat consisting of habitat patches (100×100×15 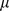
m) connected by corridors (50×5×15 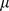
m). (**b**) Schematic diagram of a microhabitat consisting 85 connected habitat patches. Circles depict the inlet ports where bacteria are inoculated. The zoom-in shows an image of fluorescently labeled WT (green) and GASP (red) *E. coli*. The white bar indicates 50 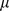
m. (**c**) Kymograph of a typical WT-GASP competition experiment in a microhabitat. Space is depicted horizontally and time vertically, each pixel represents a habitat patch which is color-coded according to its WT (green) and GASP (red) occupancy. Yellow pixels indicate the presence of WT and GASP bacteria in the same patch, the intensity of the colors scales linearly with the area fraction of a patch that is occupied.

**Figure 4:**
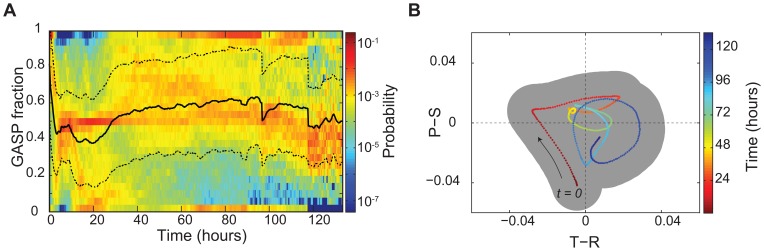
WT and GASP coexist in microhabitats. (**a**) Probability density of the GASP fraction of patches (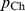) through time calculated from the occupancy data of all microhabitat experiments (a total of 1700 patches in 20 independent 85-patch microhabitats, see Fig. S1 for all data), the solid black line indicates the mean GASP fraction, dashed lines depict the mean 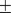 standard deviation. (**b**) Mean temporal game-dynamics of WT-GASP competition in microhabitats, the shaded area depicts the variation between experiments (standard deviation of replicates).

Microhabitats were filled with LB medium and a 1∶1 mix of WT and GASP *E. coli* was introduced into the habitat. When bacteria colonize a patchy microhabitat, the number of WT and GASP cells that establish a population in each patch, and thus the GASP fraction of patches, varies stochastically. As a consequence, many different starting GASP fractions occur in a single experiment. After the initial colonization phase, the WT-GASP community grows exponentially for 10–20 hours after which the maximum habitat-wide occupancy is reached. In the following extended stationary phase, the population size decreases but the community remains dynamic: cells remain motile, and continue to grow, divide, and die, causing the community inhabiting a patch to change continuously. The kymograph in Figure 3C, shows that population shifts occur readily at the patch level, and that the community remains dynamic for the entire duration of the experiment. At any given time, a wide variety of WT/GASP ratios occurs in different patches, as can be seen from the patch-level probability density of the GASP fraction through time (see Fig. 4A, all fractions are present at all times). On the global scale of the entire microhabitat however, the GASP fraction fluctuates around half, and cooperators and cheaters thus coexist.

To characterize the community dynamics by determining payoffs at the patch level, it is important to realize that patch-level population dynamics are not only subject to competition but are also influenced by the migration of bacteria. By grouping patches at each time point according to their GASP fraction, we can infer the competitive dynamics by analyzing the trend of the GASP fraction in each group analogous to our approach for the well-mixed cultures (see Materials and Methods). This analysis, which is not affected by random migration events, shows how patches with a similar GASP fraction change in population composition over time and enables us to estimate payoffs at the patch level (see Fig. S3). Figure 4B shows that the trajectory of the payoffs obtained by this analysis differs markedly from that of the well-mixed cultures: the 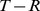 versus 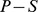 curve fluctuates around the origin. This indicates that the payoffs are effectively neutral when compounded at the patch level, and as a result WT and GASP *E. coli* coexist.

**Figure 5:**
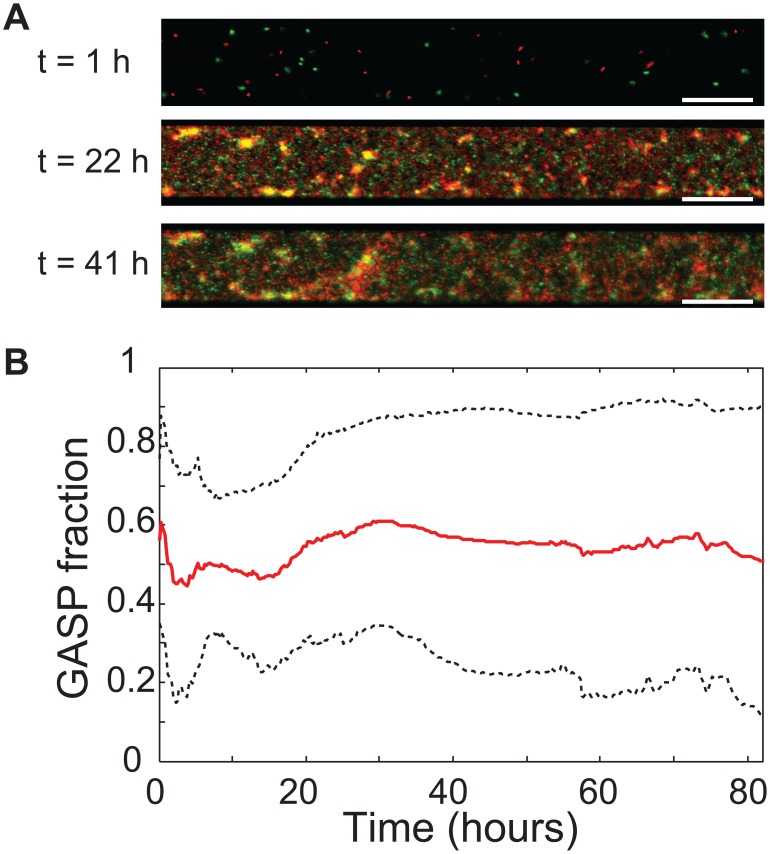
WT and GASP *E. coli* coexist in a single-patch habitat. (**a**) Microscopy pictures showing a section of a habitat consisting of a single patch (8500×100×15 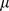
m) at three time-points, the white bar indicates 50 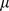
m. WT (green) and GASP (red) cells develop into a structured community. Initially (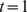 hour) planktonic cells colonize the habitat, a day later many multi-cellular aggregates have formed. The composition of these aggregates (indicated by their color) changes over time. (**b**) GASP fraction (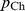) through time of microhabitats consisting of a single patch with a volume equal to the total volume of an 85-patch microhabitat. Mean of 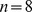 experiments (red solid line), black dashed lines indicate the mean 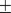 the standard deviation.

Coexistence of WT and GASP cells was observed even *within* a single patch. This raises the question whether the patchiness of the microhabitats is a prerequisite for WT-GASP coexistence: does the imposed patchiness induce a population structure that facilitates coexistence? In order to investigate this, we performed experiments in microhabitats (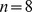) consisting of a single large patch with a volume equal to the total volume of the 85-patch microhabitat. The results in Fig. 5 show that patchiness is not a prerequisite for cooperators to persist: long-term community dynamics in a microhabitat consisting of a single large patch are identical to the habitat-wide dynamics of an 85-patch microhabitat, the GASP fraction fluctuates around 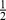. Figure 5A shows that WT and GASP cells do not distribute homogeneously through the habitat, but self-organize into a structured community consisting of planktonic cells as well as multi-cellular aggregates up to tens of micrometers in size. The composition of these multi-cellular aggregates varies, as some mainly consist of WT cells (green in Fig. 5A), others are dominated by GASP cells (red in Fig. 5A), while yet others consist of approximately the same number of WT and GASP bacteria (yellow in Fig. 5A). The presence of yellow (mixed) and red and green (pure) clusters illustrates that WT and GASP cells coexist and segregate dynamically at small spatial scales.

WT and GASP *E. coli* have previously been shown to coexist in heterogeneous landscapes in which resource availability and habitat renewal varied through space [42], [43]. Those exploratory studies suggested that WT and GASP *E. coli* spatially segregate by occupying habitat patches of different quality (e.g. differences in access to resources). Our results however, reveal that extrinsically imposed spatial heterogeneities are *not* required for coexistence of WT and GASP cells. Even in a homogeneous microfabricated habitat, where patchiness is not inherent in the spatial structure, a spatially heterogeneous community of coexisting WT and GASP cells arises. It is likely that surface growth and attachment is important for the formation of multi-cellular aggregates and thus facilitates self-structuring of the WT-GASP community. Hence, surface growth and attachment may have a key role in supporting coexistence of WT and GASP *E. coli*.

Experimental studies addressing the influence of spatial structure on cooperation often introduce structure by imposing propagation and dispersal regimes on unstructured subpopulations [3], [44], [45], while leaving little room for microbes to self-structure their environment by regulating dispersal phenotypes. Experiments conducted in biofilm flow-cells on the other hand, allow for a more natural development of population structure but flush out the vast majority of planktonic cells [15], [46]. The microhabitats utilized in this study provide a habitat landscape in which community self-assembly proceeds without artificial interventions and allows planktonic and sessile lifestyles to co-occur, as is often the case in natural habitats. As such, our study provides a valuable experimental perspective on the ecology of microbial cooperation.

## Conclusion

Understanding how cooperation may have emerged and is maintained presents a major challenge [47]: how can cooperative organisms thrive when they are so vulnerable to undermining by cheaters? This social dilemma is often interpreted in the framework of game theory, where many theoretical studies have suggested that spatial structure may have a crucial role in maintaining cooperation. In this study, we developed a game-theory based approach to quantitatively analyze community dynamics in a changing environment. By tracking WT-GASP community dynamics in various habitats, we demonstrated that in an unstructured batch culture WT cooperators and GASP cheaters eventually interact according to a prisoner’s dilemma leading to GASP dominance. By contrast, these two strains coexist when co-inhabiting a spatially structured habitat. Our findings show that surface growth and attachment allow cells to form multi-cellular aggregates and develop a heterogeneous community in a spatially structured, but otherwise homogeneous, habitat. Even in spatially-structured microhabitats lacking patchiness or spatial heterogeneities in resource availability, a self-structured community emerges in which cooperators persist.

## Materials and Methods

### Strains and Growth Conditions

All strains used in this study have been described before in Ref. [42]. Strains JEK1036 and JEK1037 carry the WT *rpoS* allele and are *Escherichia coli* W3110 lacYZ::GFPmut2 (labelled with green fluorescent protein) and W3110 lacYZ::mRFP (labelled with red fluorescent protein), respectively. JEK1032 (green GASP) and JEK1033 (red GASP) have the GASP phenotype and carry the *rpoS819* allele (described in Ref. [28]) and the same fluorescent markers and genetic background as strains JEK1036 and JEK1037, respectively. Prior to all experiments cells were taken from a 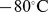 glycerol stock and grown overnight (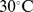, 200 rpm) in lysogeny broth (LB). GASP mutants have previously been reported to typically arise after 10 days of batch culture [9], [28], [29]. Well-mixed flask and microhabitat experiments lasted up to 7 days, experiments were not performed for longer times to prevent *de novo* mutations leading to the GASP phenotype from occurring.

### Growth Curves

Pure cultures of JEK1036 (WT) and JEK 1033 (GASP) were grown overnight in LB medium containing 100 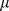
M isopropyl 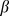-D-1-thiogalactopyranoside (IPTG). Overnight cultures were back diluted 100 times in LB medium and grown to an optical density of 0.5. These two cultures were mixed at appropriate proportions resulting in a cultures having 0%, 1%, 3%, 10%, 50%, 90% and 100% WT/GASP ratio. 100 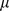
L of the mixtures was added to 300 mL LB medium containing 100 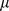
M IPTG. These cultures were grown at 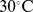 in 500 mL Erlenmeyer flasks (with aerating caps) shaking at 140 rpm. Samples of 1 mL were taken from the cultures at regular time intervals. The optical densities at 600 nm were measured after a 2X dilution with LB medium containing 100 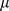
M IPTG in 1 cm plastic cuvettes using a WPA Biowave CO8000 cell density meter.

### Culture Viability Measurements

Pure cultures of JEK1036 (WT) and JEK 1033 (GASP) were grown in LB medium containing 100 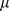
M IPTG. Overnight cultures were back diluted 100 times in LB and grown to an optical density of 0.5. These two cultures were mixed at appropriate dilutions to obtain cultures having 0%, 10%, 50%, 90% and 100% WT/GASP fractions. Six mL of these mixtures was added to 44 mL of LB medium containing 100 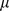
M IPTG. Three replicate cultures were made for each WT/GASP ratio. These cultures were grown at 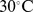 in 250 mL Erlenmeyer flasks (wit aerating caps) shaken at 140 rpm. Samples of 100 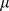
L were taken from the cultures and diluted 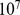 and 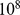 times. 100 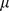
L of these dilutions were spread in replicates of 3 on LB agar plates. The plates were incubated at 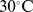 for 24 hours, and then photographed with a Nikon D5200 digital camera using a bottom illumination. Images were analyzed and colonies were counted using OpenCFU software [48].

### Flow-cytometry of Well-mixed Cultures

Flow cytometry of competition experiments was performed using a FACScan flow cytometer (Becton Dickinson) controlled using FlowJo software. GFP was excited at 488 nm and emission was filtered using a 530/30 band-pass filter, RFP was excited at 561 nm and emission was filtered with a 590/20 band-pass filter. Overnight cultures of JEK1036 and JEK1033 were separately diluted 1/50 in fresh LB supplemented with 100 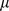
M IPTG and grown to an optical density measured at 600 nm (OD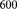) of 0.5. Three replicate cultures of 10 milliliter LB (supplemented with 100 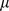
M IPTG) in 50 mL greiner tubes were inoculated with 20 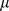
L WT-GASP mixtures with GASP fractions of respectively 0.1, 0.25, 0.5, 0.75 and 0.9, resulting in a total of 15 independent cultures. WT-GASP mixtures were made by measuring optical density of the pre-cultures and mixing appropriate dilutions at the desired ratios. Cultures were incubated for 7.5 days at 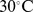 and shaken at 300 rpm. At set time intervals 30 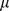
L of culture was taken, diluted in 1 mL M9 medium and vortexed for 30 seconds to be used for flow cytometry. Thirty-thousand cells were counted per sample. Events that were positive for both GFP and RFP were excluded for further analysis.

### Microhabitat Experiments

Microhabitats were fabricated in silicon following a previously published protocol [40]. The patchy microhabitats consist of 85 patches (100×100×15 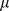
m) connected by corridors (50×5×15 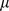
m). The dimensions of the single patch habitat are 8500×100×15 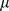
m. Two ports to inoculate bacteria were drilled through the silicon, one at each end of the habitat. Chips were closed by bonding to polydimethylsiloxane (PDMS) coated cover-slips. Microhabitats were filled with fresh LB medium supplemented with 100 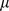
M IPTG. Overnight cultures of JEK1036 and JEK1033 (or JEK1037 and JEK1032 in half of the experiments) were separately diluted 1/100 in fresh LB +100 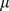
M IPTG and grown to OD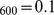. Two 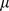
L of a 1∶1 WT:GASP mixture was pipetted in both ports which subsequently were closed using a PDMS coated cover-slip.

Microhabitats were imaged at 10 minute intervals for up to 5.5 days using an inverted Olympus IX81 microscope equipped with a 10x (N.A.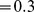) objective, an ORCA-R2 camera (Hamamatsu) and a motorized stage (Mad City Labs) controlled using 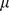
Manager software [49]. The sample was illuminated using an X-cite 120 Q (Lumen dynamics) light source. The microscope was enclosed in a home-built environmental chamber warmed to 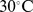.

Images were processed in MatLab using a custom script. We noticed that the two fluorescent proteins that we used (GFP and RFP) do not have the same brightness, and, more importantly react in different ways to growth phase and environmental factors like conditioning of the medium and population density. As a result, fluorescent intensity is not a good proxy for measuring biomass. To circumvent the problems associated with intensity measurements, we converted all images to occupancy data which measures the area a strain occupies. Based on the pixel values that corresponds to the autofluorescence of the medium for a certain color channel (i.e. strain), time point, and position, a threshold pixel value is calculated above which a pixel (with area 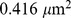) is considered to be occupied by that strain; pixels of which the intensity value is below the threshold are considered to be vacant for the strain under consideration.

### Game Theoretic Analysis

We analyzed the data of WT versus GASP competition in a game-theoretic framework. We obtained the payoffs by estimating the fitness of a single cheater cell in a cooperator majority and vice versa as outlined in the results section.

#### Well-mixed data set

We have measured the GASP cheater fraction (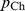) through time starting from various 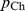 (0.1, 0.25, 0.5, 0.75, and 0.9), in triplicate. After taking the mean from triplicate data, the data was smoothed using spline smoothing in MatLab (with smoothing parameter 0.01) and numerically differentiated to obtain 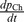 for all 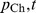 (the data from all starting fractions was used). The resulting 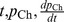 data set can be normalized by 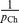 and extrapolated to 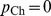 to obtain 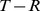 (eq. 6); or normalized by 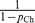 and extrapolated to 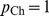 to obtain 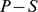 (eq. 7). Using the griddata function in Matlab, we construct a 3 dimensional surface (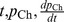) of the normalized data (see Fig. S2). Evaluating the surface normalized by 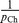 at 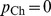, and the surface normalized by 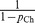 at 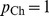, we obtain 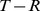 and 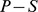. The game quadrant plot (Fig. 2B) is generated by plotting 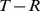 versus 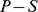. We estimated the variance in this calculation of 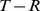 and 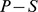 by analyzing the triplicate data sets independently as above; the shaded area around the curve in Fig. 2B and S2C represents the mean 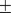 standard deviation obtained from this analysis.

#### Microhabitat data set

Patch-level population dynamics are not only subject to competition but are also influenced by (collective) migration of bacteria. In order to only analyze the competitive component of population dynamics, we group patches at each time point according to their GASP fraction in 8 equally sized bins and analyze the trend of the GASP fraction in each group. For example, all patches 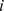 that at time 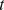 have a GASP fraction between 0 and 0.125 are assigned to bin #1 at time point 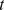. By observing the trend of the GASP fraction of a bin through time (see Fig. S3) we can see whether one of the strains has an advantage (i.e. increases in frequency) at the GASP fractions of that particular bin. As changes in GASP fraction due to e.g. differences in growth rate are much more gradual than changes due to migration, our analysis is not affected by random migration but only shows how patches with similar GASP fraction change in population composition over time. Figure S3A shows the GASP fraction versus time of all 1700 patches binned in 8 equally sized bins, for reference we have also applied the same binning to the flask data (Fig. S3B). As can be seen from Fig. S3A the community composition within a bin stays rather constant. If, for example, GASP bacteria would have a competitive advantage in patches with a small 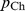, this would make 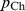 in those patches rise and result in a positive slope for the lines representing bins 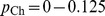 (bottom blue line) and 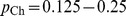 (bottom green line) as can be seen in the binned flask data. After binning the data from all microhabitat experiments (all 1700 patches) in our dataset according to their GASP fraction, we can analyze the competitive dynamics between the strains analogous to our approach for the flask data.

The surfaces obtained using this approach, shown in Fig. S4A and B, are nearly flat; except at the edge where the normalization (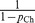 in (A) and 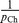 in (B)) magnifies small fluctuations. This means that the payoff differences 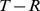 and 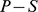 are effectively zero and competition between the strains is neutral. This can be seen in Fig. S4C which shows that the time evolution of 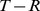 versus 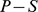 of the ensemble average of all microhabitat data fluctuates around zero. The shaded area, representing the variance in payoff differences between experiments, is obtained by generating 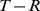 versus 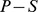 curves for all 20 microhabitats independently and depicts the corresponding standard deviation. Half the microhabitat experiments was performed with a strain pair in which WT was labeled with *GFP* and GASP with *mRFP* (strains JEK1036 and JEK1033), and half of the experiments with a strain pair in which the fluorescent markers swapped, i.e. red WT and green GASP (strains JEK1037 and JEK1032). We have performed this analysis for these two datasets separately, Fig. S4D and E show that the results of these analyses are in good agreement.

## Supporting Information

Figure S1
**Kymographs of all (** ***n*** **= 20) microhabitat experiments.** Each pixel corresponds to the GASP fraction of a single habitat patch (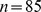 per microhabitat). Time steps (vertically) correspond to 15 minutes.(EPS)

Figure S2
**Game dynamics of well-mixed WT-GASP competition.** (**a**) Surface plot of time versus GASP fraction versus 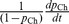, this surface can be evaluated at 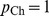 to obtain 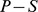. The flat green surface represents the 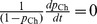 plane. (**b**) Surface plot of time versus GASP fraction versus 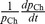, this surface can be evaluated at 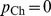 to obtain 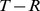. The flat green surface represents the 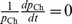 plane. To make the 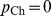 edge visible, (b) is rotated 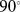 in the 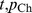 plane with respect to (a). (**c**) Time-evolution of 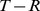 and 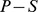 obtained by evaluating surface (a) at 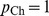 and surface (b) at 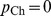, the starting point at 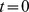 is depicted in dark red, dark blue points represent the end of the experiment at 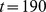 hours. We estimated the variance in the calculation of 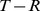 and 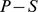 by analyzing the triplicate data sets independently as above; the shaded area around the curve represents the mean 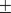 standard deviation.(EPS)

Figure S3 **Binned GASP fraction versus time.** Bin intervals are denoted on the y-axis and depicted by dashed lines. (**a**) Data from patches of all microhabitat experiments binned in 8 equally sized bins. The GASP fraction within each bin is nearly constant. (**b**) Data from the well-mixed experiments binned in 8 equally sized bins. The GASP fraction within bins changes over time.
(EPS)

Figure S4
**Game dynamics of WT-GASP competition in microhabitats.** (**a**) Surface plot of time versus GASP fraction versus 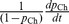, this surface can be evaluated at 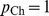 to obtain 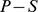. (**b**) Surface plot of time versus GASP fraction versus 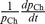, this surface can be evaluated at 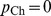 to obtain 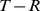. (**c**) Time-evolution of 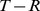 and 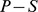 obtained by evaluating surface (a) at 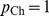 and surface (b) at 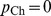, the starting point at 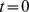 is depicted in dark red, dark blue points represent the end of the experiments at 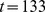 hours. The shaded area, representing the variance in payoff differences between experiments, is obtained by generating 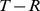 versus 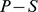 curves for all 20 microhabitats independently and depicts the mean 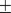 standard deviation. (**d** and **e**) Half the microhabitat experiments was performed with a strain pair in which WT was labeled with *GFP* and GASP with *mRFP* (strains JEK1036 and JEK1033), and half of the experiments with a strain pair in which the fluorescent markers swapped, i.e. red WT and green GASP (strains JEK1037 and JEK1032). We have performed the same analysis as in (c) for these two datasets separately, the results of this analysis for strains JEK1036 and JEK1033 are shown in (d) and for strains JEK1037 and JEK1032 in (e). Both datasets show similar dynamics as the pooled dataset (c), the 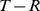 versus 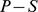 curve fluctuates around the origin.(EPS)
